# The York County Hospital: A Mother's Statement

**Published:** 1913-05-24

**Authors:** May Grant

**Affiliations:** 22 Bootham Square, York


### THE EDITOR'S LETTER-BOX.

### The York County Hospital: A Mother's Statement.

*To the Editor of* The Hospital.

Mrs. Livia Anderson Gray writes under date May 19, 1913, from The Paddocks, Acomb, York, as follows: I trust you will find room in your columns for enclosed letter. I have known Mrs. Grant for at least ten years, and have no hesitation in saying that she is an exceptionally respectable and hard-working woman. I have questioned her very closely about all the statements in this letter, and am convinced that they are all perfectly true, especially as she told me the facts at the time the child died. It was necessary for me to do the actual writing of the letter, but as far as possible I have adhered to Mrs. Grant's own words, and, as you will see, she has signed it.

---

The following is the letter enclosed:

Sir,—Before the question of the management of York Hospital is allowed to drop, I think it is only fair to give my experience of it.

A few years ago my little boy, aged six years, had to be taken to the hospital.

He had been there for three weeks, and as he seemed to be getting no better and asked me every time I saw him to take him home, I asked to have him, but was told that he was too ill to be moved. On a Sunday morning I called at the hospital at 8.30 to inquire after my child, as he was not expected to live very long. I was told that he had had a fair night and taken a little food. The nurse asked me not to go in to see him, because it upset the other children, and she promised me that should any change take place I should be sent for immediately.

At 2.30 a friend kindly said he would call at the hospital to see Arthur, and when he arrived, to his astonishment, he was told that my little boy died at 12.30, and that I might see him if I came at five o'clock. I went at once, and saw my boy in the mortuary, and then went to the undertaker, who promised to bring the body home that night, but when he reached the hospital he was not even allowed to measure the child.

On Monday at 9 a.m. I went for his death certificate, and was told the sister of the Victoria Ward wanted to see me. I accordingly went to the ward, and she said the doctor who had charge of the case wished, with my permission, to hold a post-mortem examination. I declined to allow it, and again asked for the death certificate, which they refused to give me until five o'clock.

A neighbour who was with me at the time asked the sister if my little boy had not wanted anyone, and she replied, "Oh, yes; from eleven o'clock until 12.30 he was constantly calling, 'Mother, mother.'" I may mention that the hospital is only ten minutes' walk from my house, and if I live to be one hundred years old I shall never forget that my little boy died asking for me, and that the people in charge were either too hard-hearted or too lazy to send for me.

That is my experience of York Hospital.—Yours truly,

(Mrs.) May Grant.

22 Bootham Square, York, May 19, 1913.

